# STRESSFUL LIFE EVENTS ARE ASSOCIATED WITH SELF-REPORTED FATIGUE AND DEPRESSIVE SYMPTOMS IN PATIENTS WITH MILD TRAUMATIC BRAIN INJURY

**DOI:** 10.2340/jrm.v56.13438

**Published:** 2024-03-04

**Authors:** Kaisa MÄKI, Taina NYBO, Marja HIETANEN, Antti HUOVINEN, Ivan MARINKOVIC, Harri ISOKUORTTI, Susanna MELKAS

**Affiliations:** 1 Neuropsychology; 2 Neurology, University of Helsinki and Helsinki University Hospital, Helsinki, Finland

**Keywords:** depressive symptoms, fatigue, mild traumatic brain injury, stressful life events

## Abstract

**Objective:**

To examine the associations between recent stressful life events and self-reported fatigue and depressive symptoms in patients with mild traumatic brain injury.

**Design:**

Observational cohort study.

**Participants:**

Patients (aged 18–68 years) with mild traumatic brain injury (*n* = 99) or lower extremity orthopaedic injury (*n* = 34).

**Methods:**

Data on stressful life events and self-reported symptoms were collected 3 months post-injury. Stressful life events in the last 12 months were assessed as part of a structured interview using a checklist of 11 common life events, self-reported fatigue with Barrow Neurological Institute Fatigue Scale, and depressive symptoms with Beck Depression Inventory – Fast Screen.

**Results:**

Median number of stressful life events was 1 (range 0–7) in the mild traumatic brain injury group and 1.5 (range 0–6) in the orthopaedic injury group. The groups did not differ significantly in terms of fatigue or depressive symptoms. In the mild traumatic brain injury group, the total number of recent stressful life events correlated significantly with self-reported fatigue (r_s_ = 0.270, *p* = 0.007) and depressive symptoms (r_s_ = 0.271, *p* = 0.007).

**Conclusion:**

Stressful life events are associated with self-reported fatigue and depressive symptoms in patients with mild traumatic brain injury. Clinicians should consider stressful life events when managing patients who experience these symptoms, as this may help identifying potential targets for intervention.

LAY ABSTRACT

Fatigue and depressive symptoms are common after mild traumatic brain injury. Many factors can contribute to experiencing these symptoms. This study investigated whether recent stressful life events are associated with patients’ self-reports of fatigue or depressive symptoms 3 months after having sustained a mild traumatic brain injury. It was found that recent stressful life events are connected with patients’ experience of both these symptoms. These findings suggest that healthcare professionals should ask their patients about stressful life events, as this may help in treatment planning and symptom management.

Mild traumatic brain injury (mTBI) is a major public health concern, resulting in an estimated 100–300 hospital-treated cases per 100,000 persons per year worldwide (1, 2). Although most patients with mTBI recover fully within 3 months, a substantial minority continues to experience symptoms persisting beyond this typical recovery period (3, 4).

One of the most common persistent self-reported symptoms following mTBI is fatigue (5–7). Fatigue is a subjective experience, defined by Aaronson et al. (8) as “an awareness of a decreased capacity for physical or mental activity due to a perceived imbalance in the availability, utilization or restoration of energy that is needed to perform activities”. Post-mTBI fatigue is a disabling symptom, restricting daily functioning, social participation, and ability to work (9, 10).

Persistent post-mTBI fatigue is a complex and heterogeneous phenomenon that remains poorly understood. Traditional primary injury severity measures, including presence or length of loss of consciousness (LOC), post-traumatic amnesia (PTA), or neuroimaging findings, have no clear association with fatigue (5, 9, 11, 12). Also, the role of demographic factors in post-mTBI fatigue remains inconclusive (13). Depressive symptoms and post-mTBI fatigue are closely connected and these 2 partially intertwined conditions have a complex, possibly bidirectional, relationship (13). Identifying more potential correlates for persistent self-reported fatigue is essential to improve clinical mTBI management and to allow the development of targeted, individualized treatment plans.

Stressful life events (SLEs) are experiences that disrupt individual’s usual activities, lead to considerable temporary or permanent life changes, and necessitate readjustment (14). These events include traumatic events involving life threat, such as exposure to violence or natural disasters, as well as more common events, such as losing a job or gaining a new family member (15). Both of these types of SLEs relate to adverse mental health outcomes (16, 17), and some reports suggest that they may also contribute to fatigue in several clinical groups, including chronic fatigue syndrome (18), multiple sclerosis (MS) (19), HIV (20) and ischaemic stroke (21).

As for mTBI, studies on SLEs have focused on individuals at high risk of extreme violent trauma exposure. Reid et al. (22) found that, for United States (US) service members with mTBI, exposure to life-threatening SLEs increases the risk of post-concussion and post-traumatic stress symptoms. In a civilian context, another study (23) has discovered an association between life-threatening SLEs and adverse mental health outcomes for individuals with mTBI and socioeconomically disadvantaged background. SLEs more commonly encountered in other civilian mTBI populations remain relatively unexplored, although some reports suggest that they may contribute to a worse overall symptom experience (3, 4). Data on self-reported fatigue or depressive symptoms specifically has not, to the best of our knowledge, been reported previously.

The current study examined whether recent SLEs relate to self-reported fatigue or depressive symptoms in a prospectively recruited sample of adult civilian patients with mTBI. To consider the non-specific effects of experiencing injury, the study also included an orthopaedic injury (OI) comparison group.

## METHODS

### Setting and participants

This study uses data from a cohort of patients with mTBI recruited from the Traumatic Brain Injury Outpatient Clinic of Helsinki University Hospital, Finland, from March 2015 until September 2018 (24). This unit receives referrals from Helsinki University Hospital and city hospital emergency departments (EDs), and it provides screening and evaluation of further outpatient needs for patients with TBI. The study protocol was approved by the Helsinki University Hospital Ethics Committee of Medicine. All participants provided written informed consent according to the Declaration of Helsinki.

Patients with mTBI were working aged adults (age 18–68 years) and they were enrolled in the study within 12 days after sustaining injury. Diagnosis of mTBI was based on the World Health Organization Collaborating Centre Task Force on Mild Traumatic Brain Injury criteria (25), which include 1 or more of the following: (*i*) confusion or disorientation, loss of consciousness for 30 min or less, post-traumatic amnesia less than 24 h, and/or other transient neurological abnormalities, such as focal signs, seizure, and intracranial lesion not requiring surgery; and (*ii*) Glasgow Coma Scale score of 13–15 after 30 min or later upon presentation for healthcare. Exclusion criteria were previous diagnosis of schizophrenia, schizoaffective disorder, developmental disability, current alcohol or drug dependence, visual or hearing impairment, not being fluent in Finnish, and contraindication for magnetic resonance imaging (MRI). The OI comparison group comprised patients with lower extremity OI (ankle fracture) recruited from the Trauma Emergency Department of Helsinki University Hospital. For patients with OI, any suspicion of having sustained a head injury based on hospital records, patient interview, or MRI, was a criterion for exclusion. Otherwise, the inclusion and exclusion criteria were the same as for the mTBI group.

### Procedure

Information on clinical injury characteristics, including cause of injury and presence and length of LOC and PTA, and acute brain computed tomography (CT) scan results, were obtained from ED patient records at the time of the study enrolment. All patients with mTBI underwent brain structural MRI scanning (3T, Siemens Magnetom Verio, Erlagen, Germany) 3–36 (median 10) days after injury, and patients with OI as soon after recruitment as was convenient for them. Information on SLEs, self-reported fatigue, and depressive symptoms was collected as a part of an in-person assessment 3 months after injury.

### Self-report measures

*Stressful life events.* SLEs were assessed as a part of a structured interview with an 11-item checklist comprising of events derived from frequently used SLE checklists (14, 26). The SLEs assessed included: (*i*) changes in residence; (*ii*) starting a new job; (*iii*) change in job strain; (*iv*) losing a job; (*v*) major financial difficulty; (*vi*) getting married; (*vii*) gaining a new family member; (*viii*) divorce or separation; (*ix*) major illness or injury of a family member or a close friend; (*x*) death of a family member or a close friend; and (*xi*) major personal illness or injury (excluding the index injury on which study participation was based). Participants were asked to indicate whether they had experienced (yes/no) each event in the last 12 months. Individual item endorsement and total number of events reported were recorded.

*Fatigue.* The Barrow Neurological Institute Fatigue Scale (BNI-FS) (27, 28) is a self-report questionnaire designed to assess fatigue after brain injury. It consists of 10 items rated on an 8-point scale, as follows: rarely a problem (0–1); occasional problem, but not frequent (2–3); frequent problem (4–5); a problem most of the time (6–7). Responders are asked to indicate the extent to which each of the items has been a problem for them since the injury. A total score is calculated by adding all items (theoretical range 0–70).

*Depressive symptoms.* Depressive symptoms were measured with an abbreviated version of the Beck Depression Inventory – Second Edition (BDI-II) (29) specifically designed for patients with somatic medical conditions, the Beck Depression Inventory-Fast Screen (BDI-FS) (30, 31). It was chosen for the current analyses to minimize conceptual overlap between fatigue and depressive symptom measures used. The BDI-FS consists of 7 non-somatic items from the original BDI-II (sadness, pessimism, past failure, loss of pleasure, self-dislike, self-criticalness, and suicidal thoughts or wishes), thus tapping exclusively on emotional and cognitive aspects of depressive symptoms. Each item consists of 4 alternative statements. Responders are asked to endorse the one that best describes how they are currently feeling. Items are scored on a scale from 0 to 3 and summed for a total score ranging from 0–21. Higher scores reflect greater symptom severity.

### Statistical analysis

Continuous data are presented as means with standard deviations (SD) (normally distributed variables) or medians with interquartile range (IQR) and range (non-normally distributed variables), and categorical data as numbers and percentages. Group comparisons were performed using Pearson’s χ^2^ test or Fisher’s exact test for binary categorical variables, Student’s *t*-test for normally distributed continuous variables, and Mann–Whitney *U* test for non-normally distributed continuous variables. Correlations were calculated using Spearman’s rank correlations. *p*-values below 0.05 were considered statistically significant. The data were analysed with IBM SPSS Statistics for Windows version 25 (IBM Corp, Armonk, NY, USA).

## RESULTS

### Sample characteristics

**Fig. 1:**
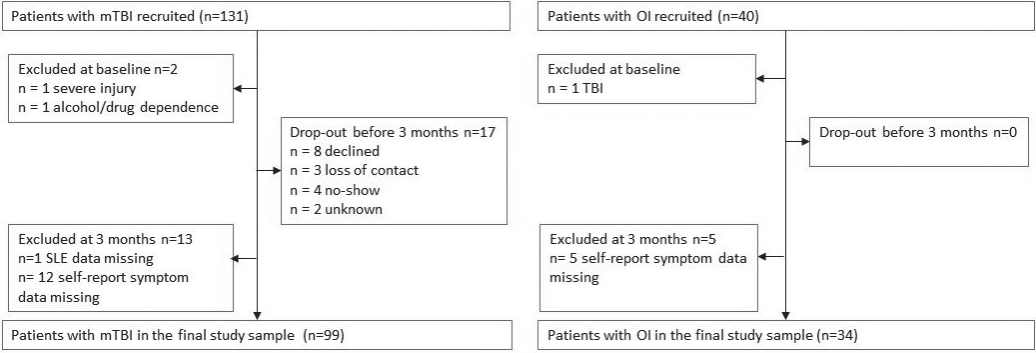
Flowchart of the study participants. TBI: traumatic brain injury; OI: orthopaedic injury.

Initially, 131 patients with mTBI and 40 patients with OI were recruited to the study. Of these, 2 patients with TBI and 1 with OI were later excluded after review of medical records or MRI revealed they did not meet the inclusion criteria. Seventeen patients with mTBI dropped out of the study before in-person assessment at 3 months, and 13 patients with mTBI and 5 with OI did not complete the measures on stressful life-events or self-reported symptoms. Thus, the final study cohort comprised 99 patients with mTBI and 34 of those had an OI (Fig. 1).

Descriptive characteristics of the final study sample are shown in Table I. The mTBI and OI groups did not differ significantly in terms of age, sex, education, or preinjury employment status. Most common cause of injury for both groups was ground-level fall. Fifty-seven patients with mTBI had a witnessed LOC (26 cases missing, *n* = 73). The duration of LOC was recorded for 51 of the 57 patients with LOC, and it was 10 min or less in all cases. Eighty-eight (88.9 %) of the patients with mTBI had PTA, and 42.4% had a trauma-related intracranial abnormality in CT (*n* = 28) or MRI (*n* = 42).

**Table I:** Demographic and injury characteristics of patients with mild traumatic brain injury and those with orthopaedic injury

Variable	mTBI (*n* = 99)	OI (*n* = 34)	Test statistic	df	*p*-value
Age, years, mean (SD)	40.3 (13.2)	42.4 (11.8)	t = –0.800	131	0.425
Sex (female), *n* (%)	49 (49.5)	17 (50)	χ^2^ = 0.003	1	1.000
Education, years, mean (SD)	15.8 (3.4)	15.9 (3.4)	t = –0.248	131	0.805
Working / full-time student prior to injury, *n* (%)	90 (90.9)	33 (97.1)			0.451^a^
Cause of injury, *n* (%)					
Motor vehicle accident	7 (7.0)	0 (0)			
Pedestrian traffic accident	2 (2.0)	0 (0)			
Bicycle accident	24 (24.2)	2 (5.9)			
Ground-level fall	29 (29.3)	16 (47.1)			
Fall from heights	20 (20.2)	7 (20.6)			
Sports	11 (11.1)	8 (23.5)			
Other	6 (6.0)	1 (2.9)			
Injury-related CT abnormalities, *n* (%)^b^	28 (29.5)				
Injury-related MRI abnormalities, *n* (%)	42 (42.4)				
Witnessed LOC, *n* (%)^c^	57 (78.1)				
Length of LOC, min, median (range)^d^	0:01–0:10				
PTA, *n* (%)	88 (88.9)				
Length of PTA, h:min, median (range)	1:17 (0:01–24:00)				

a *p*-value is for Fisher’s exact test;

b 4 missing, *n* = 95;

c 26 missing, *n* = 73;

d 6 missing, *n* = 51; CT: computed tomography; h: hours; LOC: loss of consciousness; min: minutes; MRI: magnetic resonance imaging; mTBI: mild traumatic brain injury; OI: orthopaedic injury; PTA; post-traumatic amnesia; SD: standard deviation.

There were no significant differences between the 3 months assessment non-completers and completers in terms of demographic factors (age, sex, or education) in either the mTBI or OI groups. Regarding the primary mTBI injury characteristics, the non-completers and completers did not differ significantly in duration or length of LOC or PTA, or proportion of patients with trauma-related CT findings. However, non-completers were more often MRI-negative than were completers (80 % vs 58 %, χ^2^ = 4.955, *p* = 0.031).

### Stressful life events

Median number of SLEs reported was 1 (IQR 0–2, range 0–7) in the mTBI group and 1.5 (IQR 0–2, range 0–6) in the OI group. The most common event experienced was change in job strain being reported by 37.4% and 35.3% of patients in mTBI and OI groups, respectively. The 2 groups did not differ in terms of total number of events reported or endorsement of any individual events (see Table II). Younger age was associated with reporting more SLEs (r_s_ = –0.258, *p* < 0.01) in the mTBI group. Otherwise, demographic factors were not significantly associated with SLE reporting in either group.

**Table II:** Endorsement of stressful life events in patients with mild traumatic brain injury and those with orthopaedic injury

Individual SLE endorsement	mTBI *n* = 99	OI *n* = 34	*p* ^a^
	*n* (%)	*n* %	
Change in residence	20 (20.2)	9 (26.5)	0.475
Starting a new job	16 (16.2)	8 (23.5)	0.438
Change in job strain	37 (37.4)	12 (35.3)	1.000
Losing a job	6 (6.1)	1 (2.9)	0.677
Major financial difficulty	10 (10.1)	3 (8.8)	1.000
Getting married	3 (3.0)	1 (2.9)	1.000
Gaining a new family member	1 (1.0)	1 (2.9)	0.447
Divorce or separation	10 (10.1)	5 (14.7)	0.531
Major illness or injury of a family member or a close friend	21 (21.2)	12 (35.3)	0.112
Death of a family member or a close friend	15 (15.2)	6 (17.6)	0.787
Major personal illness or injury	20 (20.2)	10 (29.4)	0.341

a *p*-values are for Fisher’s exact test. mTBI: mild traumatic brain injury; OI: orthopaedic injury; SLE: stressful life event.

### Fatigue and depressive symptoms

The mTBI and OI groups did not differ significantly in terms of self-reported fatigue (U = 1405.5, *p* = 0.150) or depressive symptoms (U = 1591.0, *p* = 0.586). As shown in Table III, the fatigue (BNI-FS) and depressive symptom (BDI-FS) total scores were significantly intercorrelated in the mTBI group. A roughly comparable positive correlation was also detected for the OI group, this did not, however, reach statistical significance. In the mTBI group, the total number of recent SLEs correlated significantly with self-reported fatigue and depressive symptom. Adjusting for age did not change these associations in any significant way. Exploration of the individual SLEs showed that patients with mTBI who had experienced major personal illness or injury in the past year experienced more fatigue (U = 523.5.0, *p* = 0.018) and depressive symptoms (U = 566.0, *p* = 0.024) compared with those not endorsing this SLE. In addition, job loss (U = 150.5, *p* = 0.019) and major financial difficulties (U = 275.0, *p* = 0.022) related to higher depressive symptom total scores. There were no statistically significant associations between SLEs and self-reported fatigue or depressive symptoms in the OI group (see Table III).

**Table III:** Self-reported symptoms and their associations (Spearman’s correlations) with stressful life events in patients with mild traumatic brain injury and those with orthopaedic injury

	mTBI *n* = 99					OI *n* = 34				
	Median	IQR	Range	BDI-FS r_s_ (*p*)	SLEs r_s_ (*p*)	Median	IQR	Range	BDI-FS r_s_ (*p*)	SLEs r_s_ (*p*)
Fatigue (BNI-FS)	5.5	0–15	0–50	0.423 (< 0.001)	0.270 (0.007)	9.5	3–14	0–51	0.304 (0.08)	–0.177 (0.389)
Depressive symptoms (BDI-FS)	0	0–1	0–11		0.271 (0.007)	0	0–1	0–6		0.153 (0.316)

BDI-FS: Beck Depression Scale Fast Screen; BNI-FS: Barrow Neurological Institute Fatigue Scale; IQR: interquartile range; mTBI: mild traumatic brain injury; OI: orthopaedic injury; r_s_: Spearman correlation r; SLEs: stressful life events.

Age, sex, or education, were not associated with fatigue or depressive symptoms in either group (all *p*-values > 0.05).

There were no significant associations between the duration of LOC and self-reported fatigue (r_s_ = –0.076, *p* = 0.595) or depressive symptoms (r_s_ = –0.130, *p* = 0.363). The duration of PTA did not correlate with self-reported fatigue (r_s_ = –0.085, *p* = 0.428) or depressive symptoms (r_s_ = –0.019, *p* = 0.860). MRI-positive and MRI-negative patients did not differ significantly from each other in terms of self-reported fatigue (U = 1018.5, *p* = 0.202). However, MRI-positive patients reported more depressive symptoms compared with MRI-negative patients (Mann–Whitney *U* = = 0.20, *p* = 0.031).

## DISCUSSION

These findings indicate that the accumulation of recent SLEs is associated with higher levels of self-reported fatigue and depressive symptoms in patients with mTBI.

While the association between SLEs and fatigue has been previously demonstrated in other clinical populations, this study is, to the best of our knowledge, the first to report a similar association in patients with mTBI. Exposure to extreme life-threatening SLEs is a known risk factor for psychological distress in patients with mTBI (22, 23), but our findings add to the existing evidence by suggesting that also more common recent SLEs may associate with post-mTBI depressive symptoms.

Of the individual SLEs explored, the experience of major personal illness or injury was associated with higher levels of fatigue and depressive symptoms in the mTBI group. This is hardly surprising, considering fatigue itself is a common symptom in various medical conditions, and reciprocal connections between physical and mental health are well established. In congruence with others’ reports, experiencing job loss or financial difficulty was associated with depressive symptoms (32, 33).

In the current study, the total number of recent SLEs was associated with self-reported fatigue and depressive symptoms in patients with mTBI, but not in those with OI. Considering the small size of the OI group, and the fact that the positive correlation between the total number of SLEs and depressive symptoms was roughly equivalent in the 2 study groups, this finding may be partly explained by lack of power. However, it is noteworthy that the correlation between SLEs and fatigue were low and negative in the OI group, while being positive in the mTBI group. This finding suggests that patients with mTBI may be specifically susceptible to experiencing fatigue in relation to accumulation of SLEs, perhaps due to increased need for compensatory effort brought about by lingering cognitive difficulties (34). To clarify this issue, however, further study is necessary.

In accordance with many previous reports (9, 10, 12), primary mTBI injury characteristics (LOC, PTA, or the presence of traumatic lesions in the MRI) were not associated with self-reported fatigue in the current study. Previous findings are mixed on whether presence of structural injury visible on neuroimaging is associated with worse emotional outcome in mTBI (12, 35–39). In the current study, patients with traumatic lesions in the MRI reported more depressive symptoms than did patients without them. This finding could reflect the physical changes or emotional response to more severe injury. It is also possible that the visible evidence of brain injury may impact patients’ appraisal of injury severity and recovery expectations, thus increasing the risk of developing depressive symptoms (40, 41).

In the current study, self-reported fatigue or depressive symptoms at 3 months did not differ significantly between the mTBI and OI groups. These findings are in line and complement the accumulating evidence that the prognosis of mTBI is generally favourable and most patients make good recovery within 3 months. However, importantly, there was a lot of variability in patients’ symptom ratings, especially regarding fatigue, illustrating the heterogeneity of mTBI recovery. Assessment of SLEs could be valuable, especially for the subgroup of patients who experience persistent post-mTBI symptoms.

The strengths of the current study include the clinically and neuroradiologically carefully examined mTBI sample and the inclusion of OI control group. Some important limitations also need to be addressed. First, the correlational study design precludes the current study from drawing any conclusions about directionality or causality. Patients with mTBI in the current study had been referred to screening of further outpatient needs, and the proportion of MRI-positive patients was high. Furthermore, patients with who did not complete the 3 months in-person assessment were more often MRI-negative than patients who completed the assessment. Thus, the final study sample is skewed towards the more severe end of the injury severity spectrum in mTBI, and the current results may not be applicable to those with milder mTBI. The sample size was relatively small, especially in the OI group, increasing the risk of type II error. While this study examined SLEs in the last 12 months, it could be beneficial to differentiate pre- and post-injury SLEs in future studies. In addition, inclusion of patient ratings of perceived significance of SLEs should be considered. Sleeping problems and physical injuries were not examined in the current study, which can be considered a weakness and should be taken into account in further studies. The fatigue measure used did not allow the current study to discern mental from physical fatigue. Finally, fatigue and depression have considerable conceptual overlap and these 2 are impossible to completely disentangle. However, to minimize the measurement overlap, we assessed depressive symptoms with the BDI-FS, which focuses exclusively on emotional and cognitive aspects of depression.

In conclusion, these findings suggest that the accumulation of recent SLEs associates with higher levels of self-reported fatigue and depressive symptoms in patients with mTBI. Clinicians should consider SLEs when managing patients experiencing persistent post-mTBI fatigue or depressive symptoms, as this may help in identifying potential targets for intervention.
